# Machine Learning Investigation of Ternary-Hybrid Radiative Nanofluid over Stretching and Porous Sheet

**DOI:** 10.3390/nano15191525

**Published:** 2025-10-05

**Authors:** Hamid Qureshi, Muhammad Zubair, Sebastian Andreas Altmeyer

**Affiliations:** 1 Department of Mathematics, Mohi-Ud-Din Islamic University, Nerian Sharif 12080, A.J.K., Pakistan; 2 Department of Mathematics, Shandong University, Jinan 250061, China; 3 Department of Physics, Castelldefels School of Telecom and Aerospace Engineering, Universitat Politècnica de Catalunya, 08034 Barcelona, Spain

**Keywords:** artificial intelligence, ternary-nano fluid velocity study, neural network, Levenberg Marquardt Feedforward Algorithm

## Abstract

Ternary hybrid nanofluid have been revealed to possess a wide range of application disciplines reaching from biomedical engineering, detection of cancer, over or photovoltaic panels and cells, nuclear power plant engineering, to the automobile industry, smart cells and and eventually to heat exchange systems. Inspired by the recent developments in nanotechnology and in particular the high potential ability of use of such nanofluids in practical problems, this paper deals with the flow of a three phase nanofluid of MWCNT-Au/Ag nanoparticles dispersed in blood in the presence of a bidirectional stretching sheet. The model derived in this study yields a set of linked nonlinear PDEs, which are first transformed into dimensionless ODEs. From these ODEs we get a dataset with the help of MATHEMATICA environment, then solved using AI-based technique utilizing Levenberg Marquardt Feedforward Algorithm. In this work, flow characteristics under varying physical parameters have been studied and analyzed and the boundary layer phenomena has been investigated. In detail horizontal, vertical velocity profiles as well as temperature distribution are analyzed. The findings reveal that as the stretching ratio of the surface coincide with an increase the vertical velocity as the surface has thinned in this direction minimizing resistance to the fluid flow.

## 1. Introduction

The application of Ternary hybrid nanofluids (THNFs) in heat transfer holds significant promise for improving the efficiency and sustainability of various manufacturing systems through extensive sort of trades. Ternary nanofluids, that blend a base fluid with nanoparticles and surfactants or stabilizers, exhibit superior thermal properties compared to the simpler, single-phase fluids, making them highly attractive for researchers. Their unique characteristics make them appropriate for numerous heat transfer applications. For instance, electronics devices like laptops, smartphones, and light emitting diode illuminations use ternary nanofluids to efficiently manage the heat formed throughout the procedure. The enhanced thermal conductivity of these nanofluids allows electronic components to function more efficiently and reliably, ensuring better heat management. Additionally, ternary nanofluids can function as a heat transfer media in intense solar power systems. These nanofluids effectively transfer absorbed solar energy to heat exchangers or storing containers, enhancing the inclusive proficiency of solar energy exchange systems. In thermal energy storage systems, the inclusion of ternary nanofluids enhances energy density and heat transfer proficiency, making them ideal for various renewable energy applications.

Various researchers [1,2,3,4,5,6] examined the thermal productivity of nanofluids. Most studies focused on the thermal capacity capabilities of these HNF and THNF. They developed novel methods, including new empirical correlations, magnetic strength applications, and enhanced thermal convection, to enhance the enthalpy maintenance of these fluids. More precisely, Fatunmbi et al. [7,8] considered the effect of activation energy on the twin stratified process in non-homogenous convection with magneto-tangent hyperbolic fluids across a stretchable plate. They applied quasi-linearization method (SQLM) to investigate dissipative flow of power-law fluids under the effects of Hall currents and power-law slip conditions when flow accompanies exponentially stretching surface.

G.K. Ramesh et al. [9,10,11] explored the 3-D Maxwell fluid flow containing on hold nanoparticles over a 2D porous and extensible surface. They further analyzed the behavior of an upper-convected Maxwell (UCM) fluid over a radiative, bi-directionally extensible plate, utilizing an innovative nonlinear Rosseland approximation for thermal radiation. Additionally, they investigated the heat transfer characteristics on a bi-directionally stretched sheet under varying thermal conditions.

Ternary nanofluids can also offer novel cooling solutions for energy-efficient infrastructure. For example, vapor compression-vapor absorption cascade refrigeration systems, which utilize geothermal, solar, and waste heat, can replace traditional single-stage vapor compression systems, offering reduced electrical consumption [12,13]. Hybridized nanofluids, with their unique thermal properties, can enhance the cooling process in industrial refrigeration, biological applications, and medical therapies, including cancer treatment and epidemic control. Carbon nanotubes (CNTs) are particularly advantageous in heat transfer applications across sectors like electronics, aerospace, thermal energy storage, fluid systems, and biomedical treatments. Carbon nanotubes consisting of substances are critical constituents in thermal exchangers, thermal capacity systems, heat limit filming and TIMs, improving heat dissipation and system performance. Mishra et al. [14] investigated the influence of radiation on the flow of Casson hybridized nanofluids over an elongating or contracting convection surface. Their study focused on the geometry of nanoparticles, specifically carbon nanotubes (CNTs), and the thermophysical properties of CNT nanofluids in kerosene oil. They examined the impacts of thermal radiative flux and magnetohydrodynamics (MHD), additionally, the interaction of radiative and magnetic forces on CNT-based nanofluids flowing over a flexible sheet in a Newtonian fluid was investigated. The works by Rafique et al. and Farooq et al. [15,16] provide a mathematical study to explore the influence of carbon nanotube (CNT) length and radius on the behavior of nanofluids over a Joule-heated surface with variable viscosity. Additionally, they examined the impact of thermal radiation on sodium alginate-consisting hybrid nanofluids in Darcy-Forchheimer model over a stretchable plate.

Thermal radiation, a phenomenon in which hot surfaces emit electromagnetic waves (mainly infrared), shows a fundamental part in heat transfer applications. The emitted photons transfer energy via absorption, LFM reflection, or transmission to surrounding surfaces. Understanding thermal radiation is vital for optimizing energy efficiency in systems like solar energy utilization, thermal regulation of electronics, insulation materials, and heating/cooling systems. Several studies [17,18,19,20], have scrutinized the impact of radiation. Expanding on these previous works, this research explores the movement of a THNF, which includes carbon nanotubes, (Au), and (Ag) nanoparticles, across a bi-directionally stretching sheet, with a focus on its application to blood flow. Various works [21,22,23,24] investigated the numerical solutions for entropy generation in flow of nanofluid over a surface, emphasizing its application to radiative management systems. They studied the influence of thermal radiation and Joule heating on the flow of a magnetized Casson sodium alginate HNF over a permeable, moveable plate. Additionally, their research explored the behavior of hybrid nanofluid flow in a vertical annulus with a porous.

Further theoretical investigation [25,26,27] have been carried out focused on the enhancement of entropy changes in mass diffusion within a 3-phase viscous fluid flowing through an inclined network. Contemporary research has delved into innumerable sides of the dynamic behavior of ternary nanofluids, a significant field of study that uncovers the intricacies of specific flow phenomena and their related impacts. Resent works [28,29] illustrated the ability of using Artificial Intelligence (AI) and Machine Learning ML with respect to predicting of buoyancy and magnetohydrodynamics for different nanofluids and hybrid nanofluids over porous media.

In this study, blood is considered as the base fluid because of its direct biomedical relevance. Lately, there have been quite a few studies focusing on blood-based nanofluids, especially for things like targeted drug delivery, hyperthermia treatments, and diagnostic uses. Metallic nanoparticles, like gold (Au) and silver (Ag), are well-known for their roles in biomedical imaging and cancer detection. On the other hand, carbon nanotubes (CNTs) have been thoroughly investigated for their applications in cancer hyperthermia and biosensing. Our mention of “cancer detection” therefore refers to the potential biomedical application of such nanofluids, rather than a direct experimental validation.

The modern AI approach, LMFA, is applied alongside a machine learning algorithm. Optimization is achieved through a stochastic technique, which aligns well with the probabilistic nature and randomness of the model under study. A purely numerical method may fall short in accurately capturing the model’s behavior compared to stochastic results. The model formulation leads to a set of PDEs, which are converted into a system of ODEs using correspondence parameters. Using AI, a dataset is generated for three scenarios, analyzing velocity and temperature profiles based on variations in key variables. This dataset is processed through 10 embedded neurons in MATLAB’s toolkit. The article compares numerical and AI-generated outcomes and discusses the errors between the two approaches. Similar techniques have been already succesfully employed on various non-linear mathematical models [30,31,32,33,34,35,36].

## 2. Materials and Methods

### 2.1. Model Development

**Figure 1:**
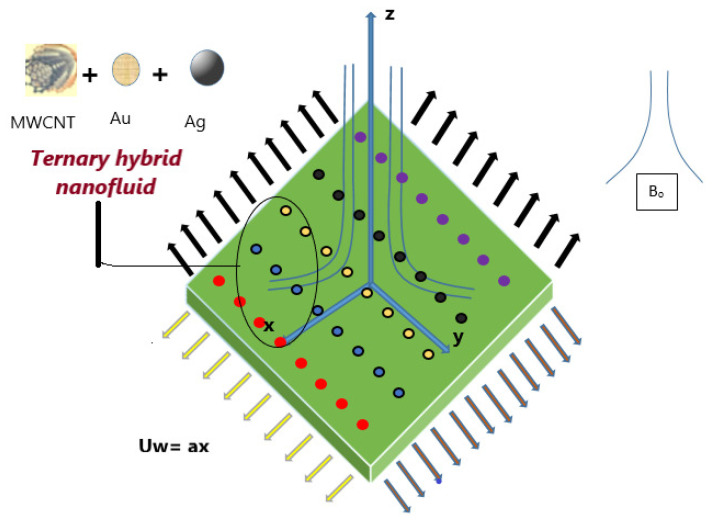
The physical arrangement of the THNF model.

In this study, we accomplish a bidirectional steady-state enquiry of a magneto-trihybrid nanofluid containing fine nanoparticles. The impact of the resistive potency (M) is incorporated into the equation of momentum to regulate the inertia of the THNF. A steady Lorentz force is applied orthogonally to the flow direction (see Figure 1). However, the resulting magnetized force is measured unimportantly in association to the functional magnetic pitch. The cscenario without any mass flux is also taken into consideration.

### 2.2. Expectations and Situations of the Framework

The following assumptions form the basis for the development of the mathematical model:

- The substance is considered porous, the single-phase (Tiwari-Das) model is used.
- The nanofluid is treated as a Newtonian fluid, with Boussinesq and boundary layer approximations applied.
- The flow exhibits thermal radiation and heat generation.
- Convective heat boundary conditions are assumed.
- Single and multi-walled Carbon Nanotubes (CNT), along with Silver (Ag) nanoparticles, are combined with plasma as the base liquid.
- The THNF is assumed to have uniformly sized, spherical nanoparticles, with no consideration for aggregation effects.

For the geometry, the surface dimensions are described using coordinates system. This one is assumed that the rate of flow along horizontal, denoted as $u_{w}$ follows the function $u_{w}=ax$, where *a* is a fixed, non-negative constant, while the velocity along vertical, denoted as $v_{w}$, follows the function $v_{w}=by$, where *b* is also a fixed, non-negative constant. The surface is preserved at a convective temperature $T_{f}$.

Throughout this manuscript, the shorthand MWCNT–Au/Ag is used to denote a ternary-hybrid nanofluid composed of multi-walled carbon nanotubes (MWCNTs), gold (Au), and silver (Ag) nanoparticles dispersed in a base fluid. This compact notation has been used in recent literature and is adopted here for brevity.

Blood has been modeled here as a Newtonian fluid following the Tiwari-Das single-phase approach. While blood exhibits shear-thinning behavior in some regimes, under moderate shear rates and dilute nanoparticle suspensions it is often approximated as Newtonian. This closure simplifies the mathematical formulation while still capturing the leading-order thermal effects of nanoparticle loading.

### 2.3. Mathematical Modeling

By using the assumptions outlined earlier, along with the standard momentum approximations, continuity, and energy equations, the governing equations for the boundary layer of THNF can be formulated [1,37].

(1) $$\begin{array}{ccc} \frac{\partial u}{\partial x}+\frac{\partial v}{\partial y}+\frac{\partial w}{\partial z} & = & 0 \end{array}$$

(2) $$\begin{array}{ccc} \left[uu_{x}+vu_{y}+wu_{z}\right] & = & \frac{\mu_{thnf}}{\rho_{thnf}}u_{zz}-\frac{\sigma_{thnf}}{\rho_{thnf}}B_{0}^{2}u-\frac{\mu_{thnf}}{\rho_{thnf}}\frac{u}{K^{*}} \end{array}$$

(3) $$\begin{array}{ccc} \left[uv_{x}+vv_{y}+wv_{z}\right] & = & \frac{\mu_{thnf}}{\rho_{thnf}}v_{zz}-\frac{\sigma_{thnf}}{\rho_{thnf}}B_{0}^{2}v-\frac{\mu_{thnf}}{\rho_{thnf}}\frac{v}{K^{*}} \end{array}$$

(4) $$\begin{array}{ccc} \left[uT_{x}+vT_{y}+wT_{z}\right] & = & \frac{k_{thnf}}{{\left(\rho c_{p}\right)}_{thnf}}T_{zz}-\frac{Q_{0}}{{\left(\rho c_{p}\right)}_{thnf}}\left(T-T_{\infty }\right)-\frac{{\left(q_{r}\right)}_{z}}{{\left(\rho c_{p}\right)}_{thnf}} \end{array}$$

The bidirectional stretching sheet is defined with $u_{w}=ax$ and $v_{w}=by$, where *a* and *b* are stretching rates in the *x* and *y* directions, respectively. Under similarity transfaormations, this leads to the boundary conditions

(5) $$\begin{array}{c} f\left(0\right)=0,f^{\prime }\left(0\right)=0,g\left(0\right)=0,g^{\prime }\left(0\right)=S=b/a,\theta^{\prime }\left(0\right)=-Bi\left(1-\theta \left(0\right)\right),\\ \;\mathrm{with}\;f^{\prime }\left(\infty \right)\to 0,g^{\prime }\left(\infty \right)\to 0,\;\mathrm{and}\;\theta \left(\infty \right)\to 0. \end{array}$$

The convective thermal boundary condition originates from the Cauchy relation, $-k_{nf}\partial T/\partial z=h\left(T_{w}-T\right)$, where *h* is the heat transfer coefficient. Upon nondimensionalization, the Biot number $Bi=h\sqrt{(\nu f/a)/kf}$ emerges naturally in the wall condition for temperature.

Radiative heat transfer is modeled using the Rosseland approximation. Expanding $T^{4}$ around $T^{\infty }$ yields $T^{4}∼4T_{\infty }^{3}T-3T_{\infty }^{4}$, which, when substituted into $q_{r}=-\frac{4\sigma^{*}}{3k^{*}}\frac{\partial T^{4}}{\partial z}$, eads to the radiation parameter $R=\frac{16\sigma^{*}T_{\infty }^{3}}{3k_{hf}}$ appearing in the reduced energy equation.

Following we explicitly show both derivations:

1. **Convective BC non-dimensionalization:** Starting with
  $$-k_{thnf}{\left.\frac{\partial T}{\partial z}\right|}_{z=0}=h\left(T_{f}-T_{w}\right).$$
  Using similarity transform, it reduces to
  $$\theta^{\prime }\left(0\right)=-\frac{k_{thnf}}{k_{f}}Bi\left[1-\theta \left(0\right)\right].$$
2. **Rosseland linearization:** Expanding $T^{4}$ about $T^{\infty }$
  $$T^{4}\approx T_{\infty }^{4}+4T_{\infty }^{3}\left(t-T_{\infty }\right).$$
  Substitution yields
  $$q_{r}=-\frac{16\sigma^{*}T_{\infty }^{3}}{3k^{*}}\frac{\partial T}{\partial z}$$
  Which leads to the modified coefficient in the energy Equation (16).

### 2.4. Investigation of the THNF Model

**Figure 2:**
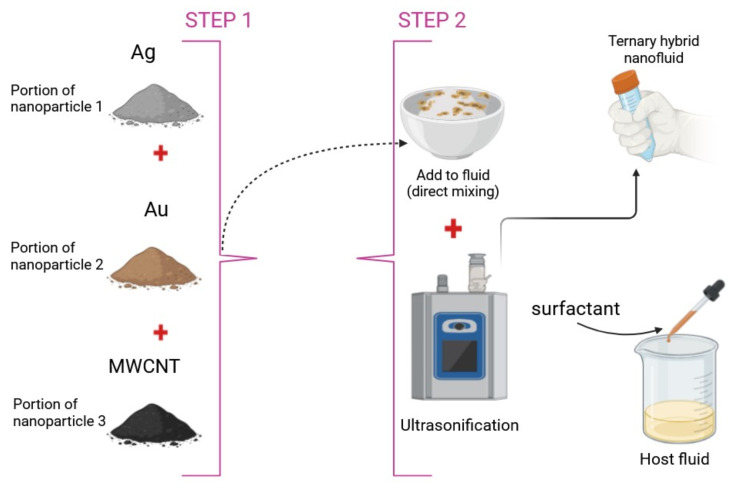
Theoretical perspective on the preparation and synthesis setup of THNF.

**Figure 3:**
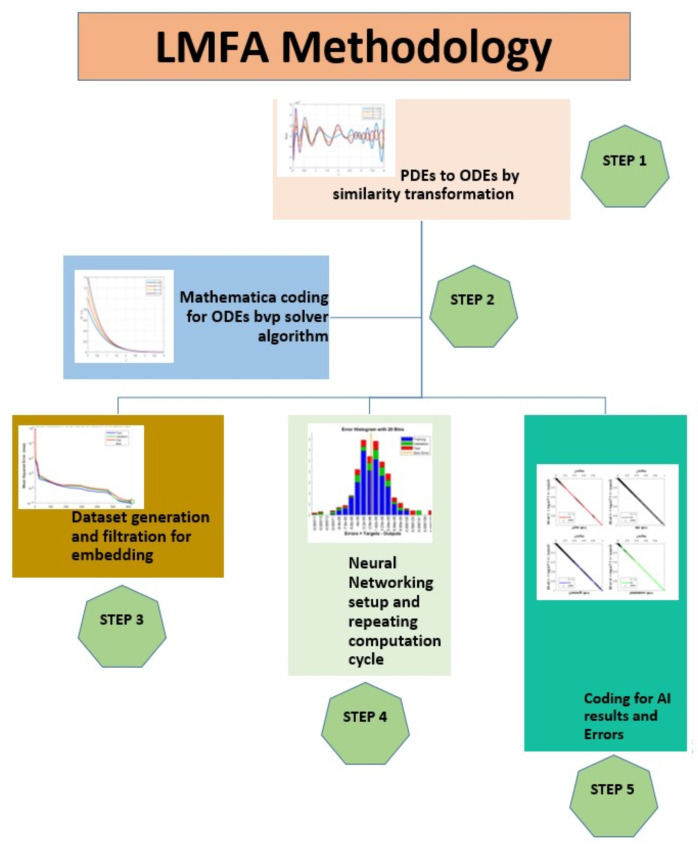
Problem Evaluation Flow Diagram.

The theoretical research and synthesis procedure of the THNF is illustrated in Figure 2. Figure 3 shows the nanoparticles of the THNF, the factors under investigation, and their thermophysical properties. The effectiveness of the thermophysical models for THNF is demonstrated in [1].

We are using,

(6) $$\frac{h_{thnf}}{u_{f}}={\left[\frac{1}{\left(1-\varphi_{1}\right)\left(1-\varphi_{2}\right)\left(1-\varphi_{3}\right)}\right]}^{\frac{5}{2}},$$

the density model

(7) $$\frac{\rho_{thnf}}{\rho_{f}}=\left(1-\varphi_{3}\right)\left[\left(1-\varphi_{2}\right)\left(1-\varphi_{1}+\frac{\rho_{s_{2}}}{\rho_{f}}\varphi_{1}\right)+\frac{\rho_{s_{2}}}{\rho_{f}}\varphi_{2}\right]+\frac{\rho_{s_{3}}}{\rho_{f}}\varphi_{3},$$

the heat capacity model

(8) $$\frac{{\left(\rho c_{p}\right)}_{thnf}}{{\left(\rho c_{p}\right)}_{f}}=\left(1-\varphi_{3}\right)\left[\left(1-\varphi_{2}\right)\left(1-\varphi_{1}+\frac{{\left(\rho c_{p}\right)}_{s_{2}}}{{\left(\rho c_{p}\right)}_{f}}\varphi_{1}\right)+\frac{{\left(\rho c_{p}\right)}_{s_{2}}}{{\left(\rho c_{p}\right)}_{f}}\varphi_{2}\right]+\frac{{\left(\rho c_{p}\right)}_{s_{3}}}{{\left(\rho c_{p}\right)}_{f}}\varphi_{3},$$

lectrical conductivity model

(9) $$\frac{\rho_{thnf}}{\rho_{hnf}}=\frac{\sigma_{s_{3}+2\sigma_{hnf}-2\left(\sigma_{hnf}-\sigma_{s_{3}}\right)\varphi_{3}}}{\sigma_{s_{3}+2\sigma_{hnf}+\left(\sigma_{hnf}-\sigma_{s_{3}}\right)\varphi_{3}}},\;\frac{\rho_{hnf}}{\rho_{nf}}=\frac{\sigma_{s_{2}+2\sigma_{nf}-2\left(\sigma_{nf}-\sigma_{s_{2}}\right)\varphi_{2}}}{\sigma_{s_{2}+2\sigma_{nf}+\left(\sigma_{nf}-\sigma_{s_{2}}\right)\varphi_{2}}},$$

thermal conductivity model

(10) $$\begin{array}{ccc} \frac{k_{thnf}}{k_{hnf}} & = & \frac{k_{s_{3}}+2k_{hnf}-2\left(k_{hnf}-k_{s_{3}}\right)\varphi_{3}}{k_{s_{3}}+2k_{hnf}+\left(k_{hnf}-k_{s_{3}}\right)\varphi_{3}},\\ \frac{k_{hnf}}{k_{nf}} & = & \frac{k_{s_{2}}+2k_{nf}-2\left(k_{nf}-k_{s_{2}}\right)\varphi_{2}}{k_{s_{2}}+2k_{nf}+\left(k_{nf}-k_{s_{2}}\right)\varphi_{2}},\\ \frac{k_{nf}}{k_{f}} & = & \frac{k_{s_{1}}+2k_{f}-2\left(k_{f}-k_{s_{1}}\right)\varphi_{1}}{k_{s_{1}}+2k_{f}+\left(k_{f}-k_{s_{1}}\right)\varphi_{1}}. \end{array}$$

The effective properties of the ternary-hybrid nanofluid are modeled using nested mixing rules, consistent with prior hybrid nanofluid studies. The density, specific heat, viscosity, thermal conductivity, and electrical conductivity are given in Equations (7)–(10). These relations assume dilute suspensions without interfacial resistance, and they reduce to the base fluid properties when $\varphi_{i}\to 0$. For clarity, all property formulas are consolidated here to aid reproducibility.

Utilizing the similarity transformation,

(11) $$u=axf^{\prime }\left(\eta \right),\;v=ayg^{\prime }\left(\eta \right),\;w=-\sqrt{av_{f}}(f\left(\eta \right)-g\left(\eta \right),\;\eta =z\sqrt{\frac{a}{v_{z}}},\;\theta =\frac{T-T_{\infty }}{T_{f}-T_{\infty }}.$$

The former Equtions (1)–(4) are transformed into:

(12) $$\begin{array}{c} f^{\prime \prime \prime }=\frac{x_{22}}{x_{11}}\left[f^{\prime 2}-\left(f+g\right)f^{\prime \prime }\right]-\frac{x_{33}}{x_{11}}M^{2}f^{\prime }-Da\;f^{\prime }=0, \end{array}$$

(13) $$\begin{array}{c} g^{\prime \prime \prime }=\frac{x_{22}}{x_{11}}\left[g^{\prime 2}-\left(f+g\right)g^{\prime \prime }\right]-\frac{x_{33}}{x_{11}}M^{2}g^{\prime }-Da\;g^{\prime }=0, \end{array}$$

(14) $$\begin{array}{c} \left(x_{55}+Rd\right)\theta^{\prime \prime }+x_{44}Pr\left(f+g\right)\theta^{\prime }+Pr\;R\;\theta =0. \end{array}$$

Thus, $f^{\prime }$ denote the horizontal velocity component, $g^{\prime }$ denote the vertica velocity component and θ the temperature distribution, respectively.

The constrains and boundary conditions are

(15) $$\begin{array}{c} f^{\prime }\left(0\right)=1,f\left(0\right)=0,g^{\prime }\left(0\right)=S,g\left(0\right)=0,\\ \theta^{\prime }\left(0\right)=-\frac{k_{f}}{k_{thnf}}B_{i}\left(1-\theta \left(0\right)\right),\\ f^{\prime }\left(\infty \right)\to 0,g^{\prime }\left(\infty \right)\to 0,\theta \left(\infty \right)\to 0, \end{array}$$

and

(16) $$\frac{u_{thnf}}{u_{f}}=x_{11},\frac{\rho_{thnf}}{\rho_{f}}=x_{22},\frac{\sigma_{thnf}}{\sigma_{f}}=x_{33},\frac{{\left(\rho c_{p}\right)}_{thnf}}{{\left(\rho c_{p}\right)}_{f}}=x_{44},\frac{k_{thnf}}{k_{f}}=x_{55},$$

The different here consideres parameters are:

- Magnetic Parameter: $M=\frac{\sigma B_{0}^{2}}{a\rho f}$
- Biot value: $B_{i}=\frac{h}{k_{f}}\sqrt{\frac{v_{f}}{a}}$
- Prandtl ratio: $Pr=\frac{v{\left(\rho c_{p}\right)}_{f}}{k_{f}}$
- Velocity Ratio Parameter: $S=\frac{b}{a}$
- Darcy numbers: $Da=\frac{v_{f}}{aK^{*}}$
- Radiation Parameter: $R=\frac{16\sigma^{*}T_{\infty }^{3}}{3k^{*}k_{f}}$
- *x*-wall stresses: $C_{f_{x}}=\frac{u}{\rho_{\infty }v^{2}/2}{\left(\frac{\partial u}{\partial z}\right)}_{z=0}$
- *y*-wall stresses: $C_{f_{y}}=\frac{u}{\rho_{\infty }v^{2}/2}{\left(\frac{\partial v}{\partial z}\right)}_{z=0}$
- Nusselt number: $Nu_{x}=-\frac{x}{T_{f}-T_{\infty }}\frac{k_{thnf}}{k_{f}}{\left(T_{z}\right)}_{z=0}+{\left(qr\right)}_{z=0}$

Applying the similarity transformation from Equation (11) one finds

(17) $$\begin{array}{c} \sqrt{Re_{x}}C_{f_{x}}=f^{\prime \prime }\left(0\right),\;\sqrt{Re_{y}}C_{f_{y}}=g^{\prime \prime }\left(0\right),\;Nu_{z}{\left(Re_{x}\right)}^{-\frac{1}{2}}=-\left(\frac{k_{thnf}}{k_{f}}+R\right)\theta^{\prime }\left(0\right), \end{array}$$

with the two Reynolds numbers $Re_{x}=\frac{ax^{2}}{v_{f}}$ and $Re_{y}=\frac{by^{2}}{v_{f}}$ in horizontal and vertical direction, respectively.

## 3. Solution Methodology and Results

In this study we introduce an innovative machine learning (ML) approach to analyze mean variability and proposes a hybrid platform for solving nonlinear (PDEs). These PDEs are applied to optimize thermal fluid dynamics in THNF flow over a stretched surface. The process begins with substituting a specific set of transforms with generalized spline expressions, adjusted using fine-tuning parameters. Next, a computational framework is developed in Python, leveraging the finite difference method to solve the resulting (ODE) system.

For reproducibility, the following numerical and AI details are provided. Governing ODEs were solved using SciPy’s solve_bvp with tolerance $10^{-8}$ on a uniform grid of 400 points; mesh refinement (200–600 nodes) confirmed domain independence at $\eta_{\infty }=10$. The AI training employed a Levenberg–Marquardt neural network (LMFA) with 70/15/15 split. Inputs were normalized via min–max scaling. The loss function was Mean Square Error (MSE); Root Mean Square Error (RMSE) and Mean Absolute Error (MAE) are reported in Table 3. Damping factor μ was initialized at $10^{-3}$ and adaptively updated. Training stopped upon achieving validation error < $10^{-6}$ or after 1000 epochs. Pseudo code, solver and scripts have been deposited at Zenodo [38] for transparency and reuse.

The ODE system was solved numerically using a finite-difference scheme in Python (SciPy). These solutions were used to generate training datasets for the Levenberg–Marquardt feed-forward neural network (LMFA). The network was implemented with 10 hidden neurons, a training/validation/testing split of 70/15/15, and error convergence down to $10^{-6}$–$10^{-9}$. This surrogate model enables efficient parametric sweeps once trained, while maintaining consistency with the baseline numerical solver.

**Figure 4:**
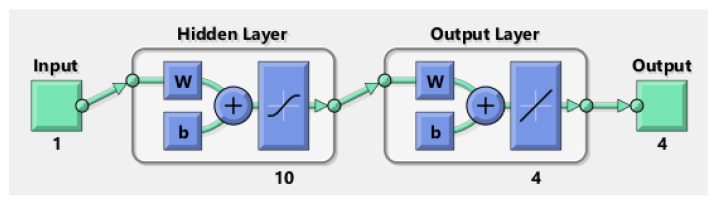
LMFA neural diagram.

**Figure 5:**
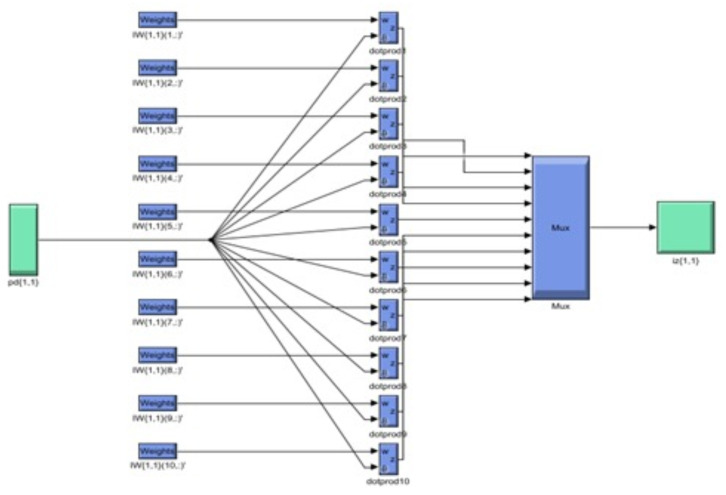
Schematics of the neural network’s internal architecture.

Python is employed to calculate velocity, temperature, and entropy for THNF and HNF cases, with results plotted and compared against AI-generated outputs. Additionally, Python facilitates data transfer to MATLAB, where the neural network model is applied. The AI technique utilizes the Levenberg-Marquardt Neural Network Algorithm (LMFA), a self-learning method. The algorithm is developed with a data partitioning strategy, allocating 70% for training, 15% for validation and testing each. In the current research we explore three factors, horizontal and vertical flow velocity, as well temperature, while we investigat the influence of five critical parameters: Magnetic parameter, velocity ratio, porosity parameter, radiation parameter and retardation factor. The neural network model comprises a 10-neuron computation layer followed by a 6-neuron output layer. The impact of three distinct values for each parameter influencing caloric and momentum change behavior across the modified wall boundary is analyzed. Figure 4 presents MATLAB-generated diagrams detailing the embedded data processing layout, while Figure 5 illustrates the neural network’s internal architecture, including embedded weight functions and the progression of epoch treatments. The parametric values used in the computations are summarized in Table 1 and Table 2, with all other variables and coefficients considered without assumptions. The nondimensional numbers utilized in the computational analysis are chosen based on various physical characteristics and conditions, providing a comprehensive understanding of system behavior. This selection enables generalization of the findings and supports the design and optimization of real-world applications. Three variations of each parameter are analyzed to observe overall trends in THNF and HNF behavior. Numerical outputs generated using AI and Python are integrated into the results section for comparison and validation.

**Figure 6:**
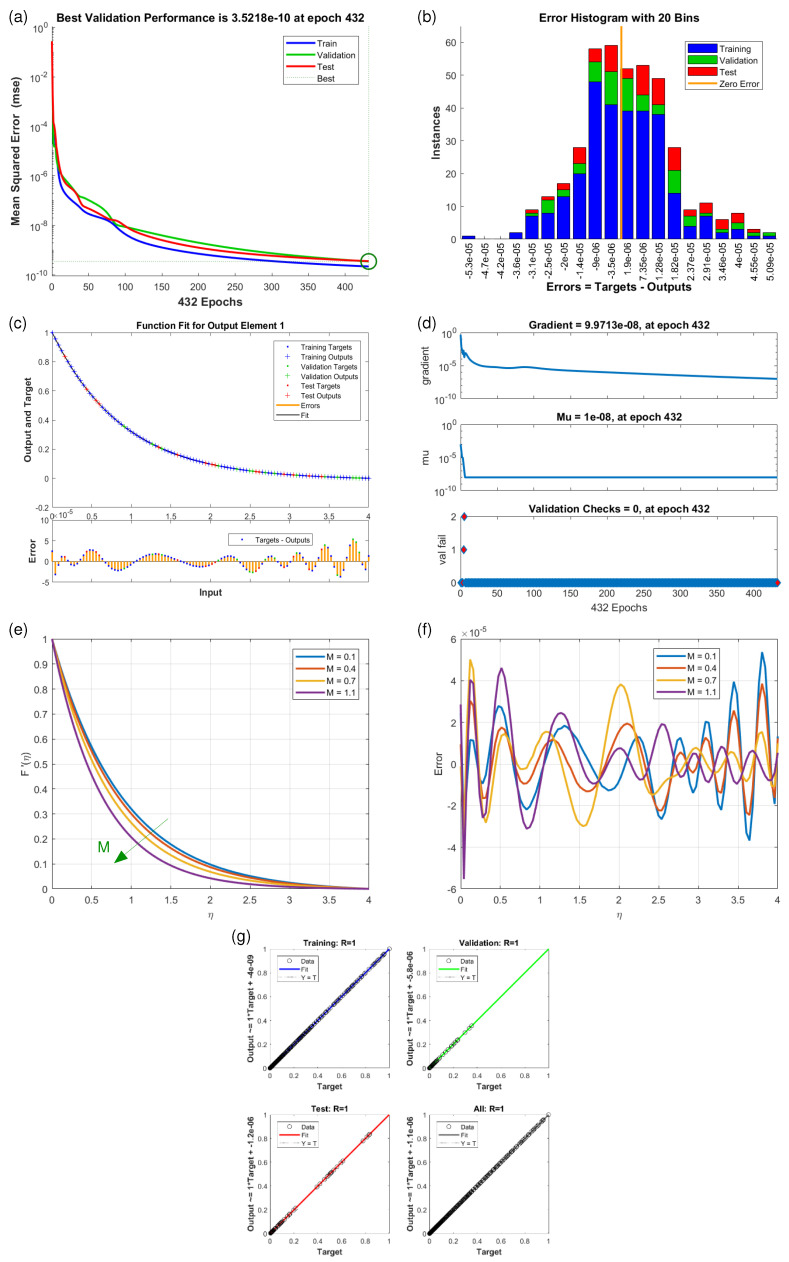
Results of horizontal velocity component $F^{\prime }\left(\eta \right)$ for scenario S-1 (Table 3) with variation of Magnetic Parameter (*M*). Shown are (**a**) Performance state for $F^{\prime }\left(\eta \right)$; (**b**) E.H for $F^{\prime }\left(\eta \right)$; (**c**) Fitness state for $F^{\prime }\left(\eta \right)$; (**d**) Training state for $F^{\prime }\left(\eta \right)$; (**e**) Solution of THNF for $F^{\prime }\left(\eta \right)$; (**f**) Error Profile of THNF for $G^{\prime }\left(\eta \right)$; (**g**) Regression Analysis of THNF for $F^{\prime }\left(\eta \right)$.

**Figure 7:**
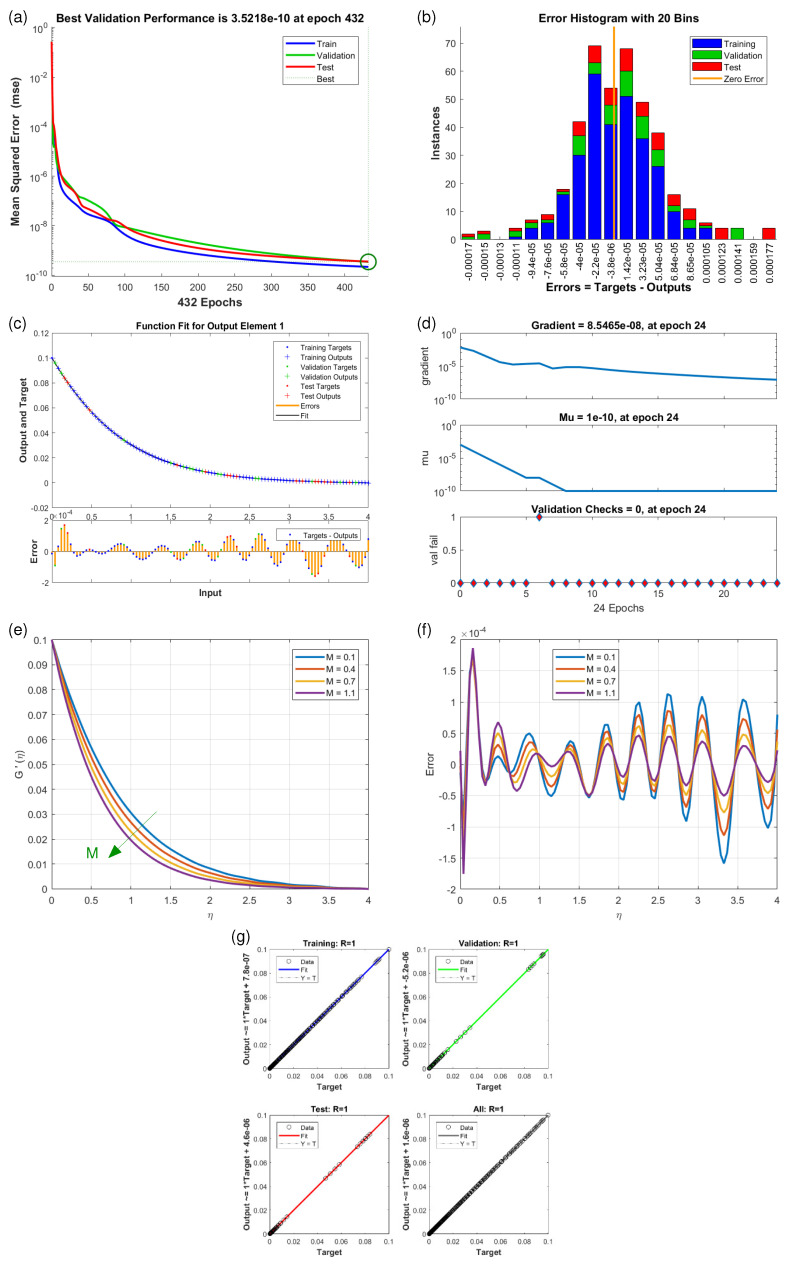
Results of vertical velocity component $G^{\prime }\left(\eta \right)$ for scenario S-2 (Table 3) with variation of Magnetic Parameter (*M*). Shown are (**a**) Performance state for $G^{\prime }\left(\eta \right)$; (**b**) E.H for $G^{\prime }\left(\eta \right)$; (**c**) Fitness state for $G^{\prime }\left(\eta \right)$; (**d**) Training state for $G^{\prime }\left(\eta \right)$; (**e**) Solution of THNF for $G^{\prime }\left(\eta \right)$; (**f**) Error Profile of THNF for $G^{\prime }\left(\eta \right)$; (**g**) Regression Analysis of THNF for $G^{\prime }\left(\eta \right)$.

**Figure 8:**
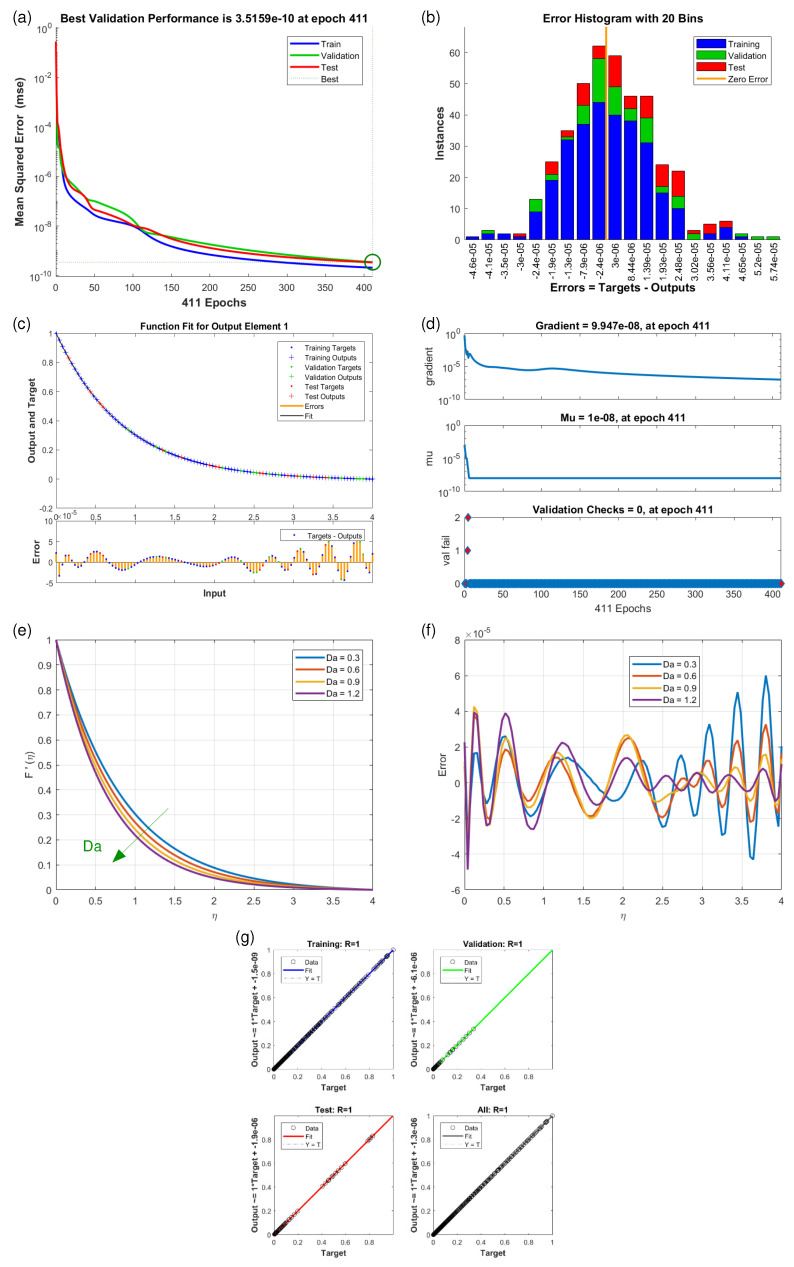
Results of horizontal velocity component $F^{\prime }\left(\eta \right)$ for scenario S-3 (Table 3) with variation of Darcy Number (Da). Shown are (**a**) Performance state for $F^{\prime }\left(\eta \right)$; (**b**) E.H for $F^{\prime }\left(\eta \right)$; (**c**) Fitness state for $F^{\prime }\left(\eta \right)$; (**d**) Training state for $F^{\prime }\left(\eta \right)$; (**e**) Solution of THNF for $G^{\prime }\left(\eta \right)$; (**f**) Error Profile of THNF for $F^{\prime }\left(\eta \right)$; (**g**) Regression Analysis of THNF for $F^{\prime }\left(\eta \right)$.

**Figure 9:**
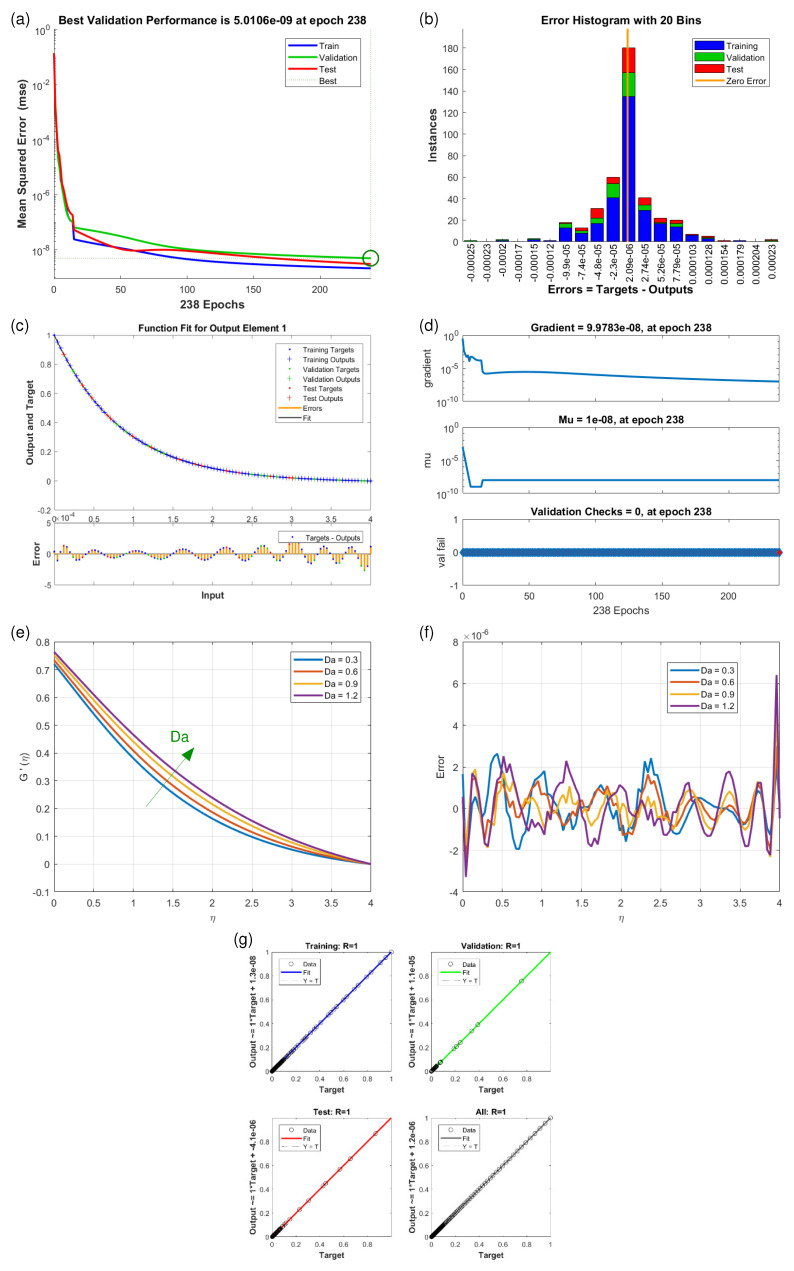
Results of vertical velocity component $G^{\prime }\left(\eta \right)$ for scenario S-4 (Table 3) with variation of Darcy Number (Da). Shown are (**a**) Performance state for $G^{\prime }\left(\eta \right)$; (**b**) E.H for $G^{\prime }\left(\eta \right)$; (**c**) Fitness state for $G^{\prime }\left(\eta \right)$; (**d**) Training state for $G^{\prime }\left(\eta \right)$; (**e**) Solution of THNF for $G^{\prime }\left(\eta \right)$; (**f**) Error Profile of THNF for $G^{\prime }\left(\eta \right)$; (**g**) Regression Analysis of THNF for $G^{\prime }\left(\eta \right)$.

**Figure 10:**
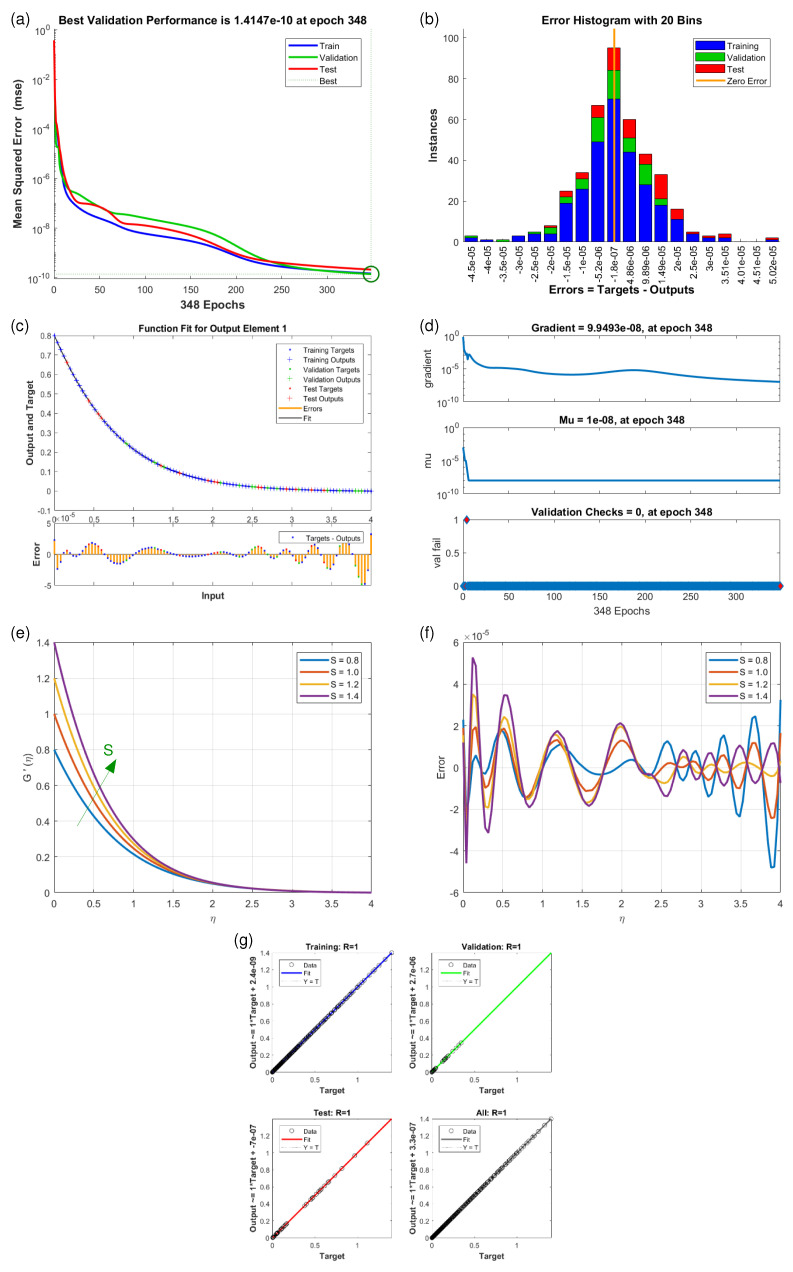
Results of vertical velocity component $G^{\prime }\left(\eta \right)$ for scenario S-5 (Table 3) with variation of Velocity Ratio Parameter (*S*). Shown are (**a**) Performance state for $G^{\prime }\left(\eta \right)$; (**b**) E.H for $G^{\prime }\left(\eta \right)$; (**c**) Fitness state for $G^{\prime }\left(\eta \right)$; (**d**) Training state for $G^{\prime }\left(\eta \right)$; (**e**) Solution of THNF for $G^{\prime }\left(\eta \right)$; (**f**) Error Profile of THNF for $G^{\prime }\left(\eta \right)$; (**g**) Regression Analysis of THNF for $G^{\prime }\left(\eta \right)$.

**Figure 11:**
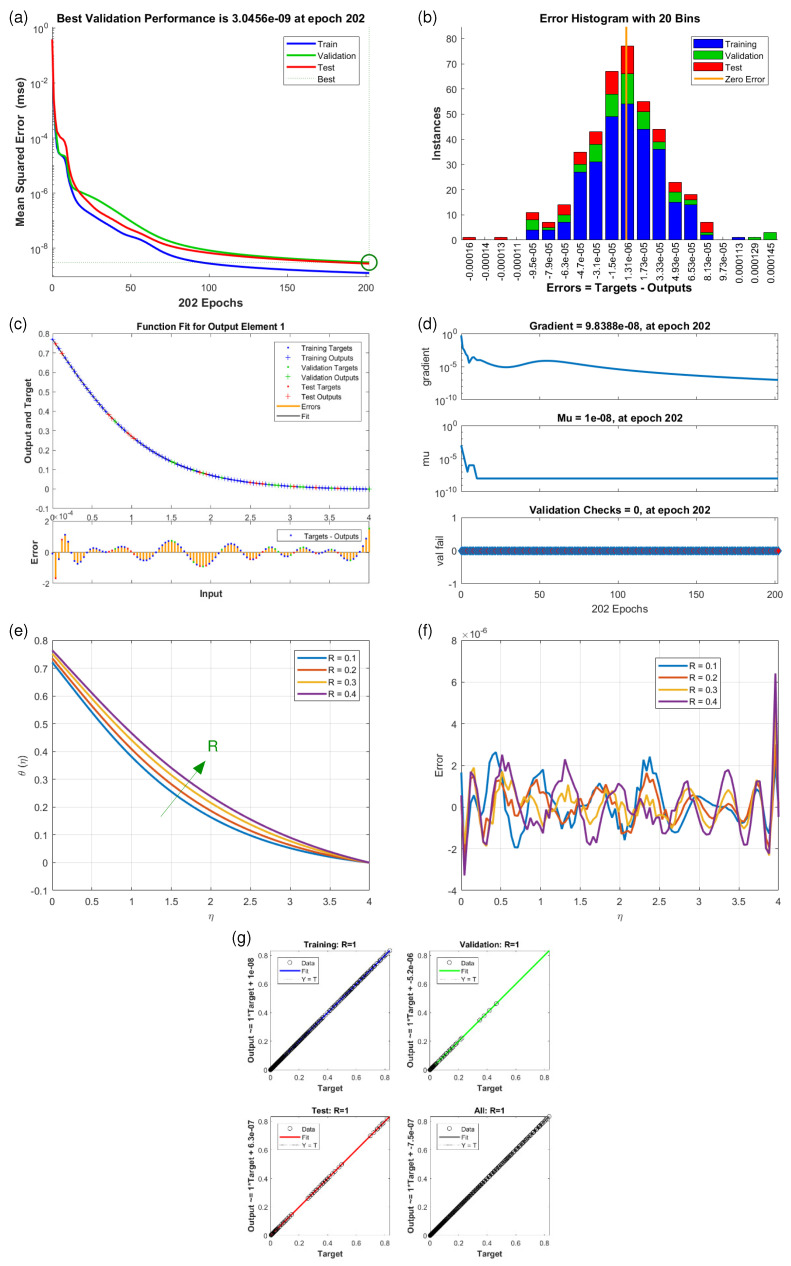
Results of θ(η) for scenario S-6 (Table 3) with variation of Radiation Parameter (*R*). Shown are (**a**) Performance state for θ(η); (**b**) E.H for θ(η); (**c**) Fitness state for θ(η); (**d**) Training state for θ(η); (**e**) Solution of THNF for θ(η); (**f**) Error Profile of THNF for θ(η); (**g**) Regression Analysis of THNF for θ(η).

**Figure 12:**
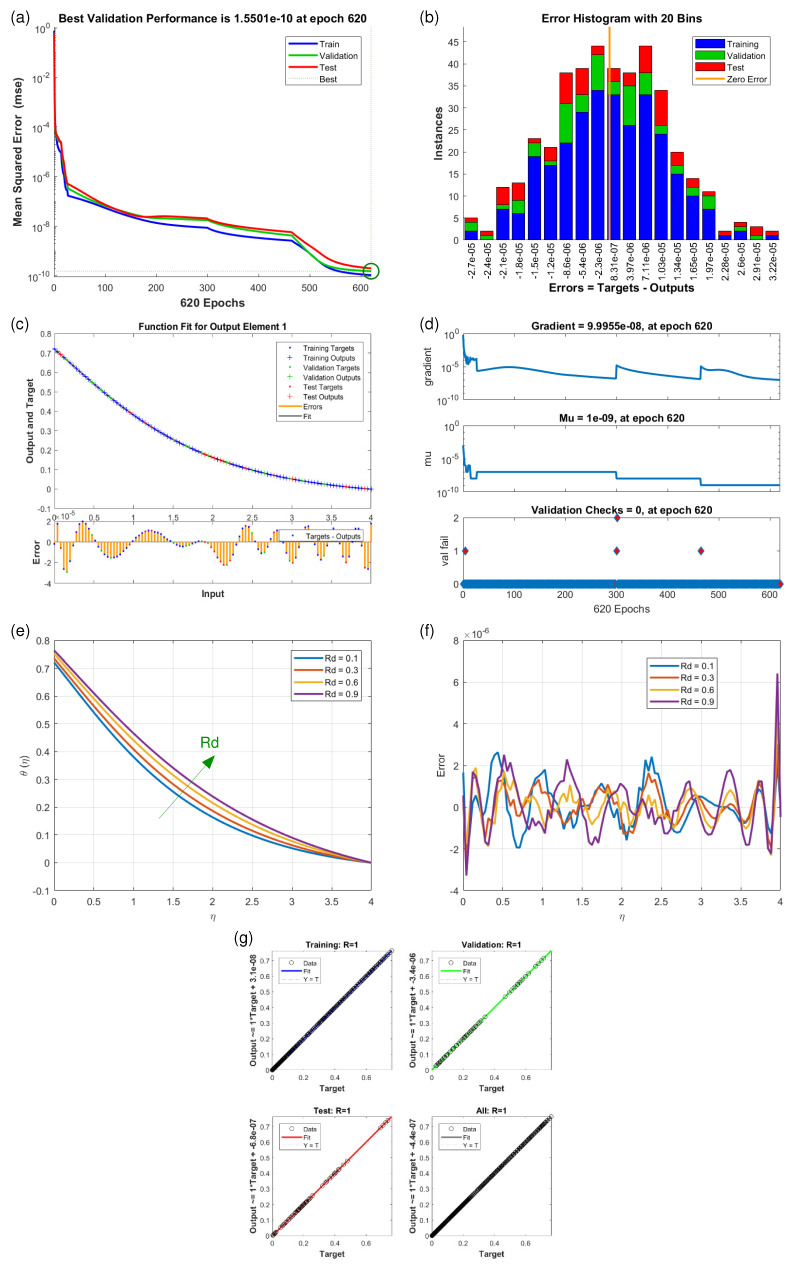
Results of θ(η) for scenario S-7 (Table 3) with variation of Retardation factor (Rd). Shown are (**a**) Performance state for θ(η); (**b**) E.H for θ(η); (**c**) Fitness state for θ(η); (**d**) Training state for θ(η); (**e**) Solution of THNF for θ(η); (**f**) Error Profile of THNF for θ(η); (**g**) Regression Analysis of THNF for θ(η).

This article presents training plots, fitness curves, error histograms, regression analysis, and performance assessments of the AI computation model, depicted in Figure 6, Figure 7, Figure 8, Figure 9, Figure 10, Figure 11 and Figure 12. The different graphs showcase the influence of essential factors, including the Casson parameter, peak point characteristics, and parameters related to transient and stretching surfaces, on flow rate. Furthermore, the temperature profile is illustrated through graphical representations of the Prandtl number.

Additionally, the variations of influencing factors are summarized in Table 3 for all scenarios.

Table 4 highlights the convergence parameters and mean square errors generated by LMFA. The method’s accuracy and precision are evident from the errors ranging from $10^{-9}$ to $10^{-10}$, along with the number of epochs and Mu and gradient grids spanning $10^{-7}$ and $10^{-9}$, respectively. The flow chart presented in Figure 3 provides a visual representation of the entire computational process.

## 4. Discussion

### 4.1. Model Validation

In order to validate the used model several consistency checks were carried out:

1. **Limiting cases:** The model recovers Newtonian single-phase blood when $\phi_{i}\to 0$.
2. Parameter realism: The chosen ranges of Bi, Pr, M, Da, and R align with experimentally and numerically reported values in biomedical and engineering contexts [31].
3. **Domain truncation:** Increasing $\eta_{\infty }$ from 10 to 15 did not affect velocity or temperature profiles, confirming domain adequacy.
4. **Dual-solution probe:** In our study, we used the Python bvp_solver to test for multiplicity by employing different initial guesses, mesh refinements (200–600 points), and extended computational domains ($\eta_{\infty }=10$ to 15). Across all parameter ranges considered (bidirectional stretching, a,b > 0), the solver consistently converged to a unique branch.

The Figure 6, Figure 7, Figure 8, Figure 9, Figure 10, Figure 11 and Figure 12 illustrate the main results of the current study. They are subdivide as follows. Subplot (a) shows the performance of training, testing and validation for THNF flow using LMNA by uzing the Mean Square Error. (b) presents the error histograms of Python-LMNA evaluation of the THNF flow. (c) gives the fitness analysis of Python-LMNA evaluation of the THNF flow. (d) presents the state transition dynamics. (e) illustrates the variation of the velocity profile $F^{\prime }\left(\eta \right)$, $G^{\prime }\left(\eta \right)$ or the temperature distribution θ(η), respectively, with variation of corresponding control parameter. (f) gives the corresponding error profile. (g) provides the regression analysis of LMNA for THNF flow over stretched porous surface.

As an internal validation, we verified that the model reduces to base fluid behavior when nanoparticle volume fractions vanish, and recovers classical nonporous, nonmagnetic, and nonradiative cases when the respective parameters are set to zero. These limiting cases confirm internal consistency of the formulation.

As mentioned before the Subplot (a) (in Figure 6, Figure 7, Figure 8, Figure 9, Figure 10, Figure 11 and Figure 12) provides an overview of the performance of the code. A comparison between testing and validation curves for profiles of velocities $F^{\prime }\left(\eta \right)$, $G^{\prime }\left(\eta \right)$ and temperature, θ(η) are shown. In any case, a clear overlapping of training, testing and validation curves is identified. Test-Training-Validation (TTV) curves are represented in blue, green and red, respectively. The best validation check is represented by a dotted horizontal line. Overlapping or parallel course of curves depicts ideal condition of training and evident for best outputs. In all cases these errors are below $10^{-4}$ and down to $10^{-6}$.

Subplots (b) in Figure 6, Figure 7, Figure 8, Figure 9, Figure 10, Figure 11 and Figure 12 represent various error histograms (EHs) in terms of mean-square errors (MSEs) for different scenarios (S-1 to S-7). These histograms plot the distributions of the MSEs in each case to obtain an idea about the variations of the errors with respect to the situations. Each histogram gives the frequency of occurrence of error values within a specific bin. The colour coding of the bars is as follow: blue for the first, red for the second, and green for the third set of TTV data. These bars identify how often certain error values occur within their corresponding bins in each TTV dataset. High bars for lower error values indicate better performance, meaning more frequent small errors, and high error values with high bars mean the reverse-worse performance, characterized by more frequent large errors. Comparing the histograms from scenarios S-1 through S-7 conveys how various aspects drive the shape of error distributions. This comparison will show which of the scenarios produce lower or higher errors and will, hence, clearly put forward, through the distribution of the MSEs, the performance of different scenarios. To guide the eyes, also a vertical yellow line is drawn at the zero-error point; the height reflects the maximum MSE as seen in the histogram. This line also serves as a point of reference since it marks the best-case scenario where the error is zero. The generated data are spread over 20 bins, which are very useful in analysing the spread and frequency of the errors. The different bars of the TTV data represent different TTV processes. The height of the different bars above indicates the number of iterations for which the error is within the range represented by that bin. The vertical axis indicates the number of iterations for a given value of the error, while the horizontal axis gives the target-output difference, which is a measure of how far the output values are from the target values ? this is something very close to the error magnitude.

Further insight for the error analysis by zooming in on the AI evaluations of the LMFA training’s fitness is provided in subplots (c) in Figure 6, Figure 7, Figure 8, Figure 9, Figure 10, Figure 11 and Figure 12. These indicate the possible representative fitness curves of the LMFA algorithm with high accuracy in velocity and temperature trend predictions. The fitness curves perfectly match the general trend and trajectory for both parameters, therefore portraying a high level of precision by the AI-generated solutions, as compared to the Python-generated solutions. None of the points on Testing, Training and Validation for these coloured crosses on the plot deviate from the trajectory and thus perfectly depict computational convergence of the LMFA. This alignment thus shows that the LMFA algorithm is very well-trained to optimal performance, producing near-minimal errors, which is shown by the closeness of the fit between the predicted values and the actual values with respect to velocity and temperature.

Finally, the overall scenario of regression analysis for TTV outputs from different processes to estimate the model performance is presented in subplots (g) in Figure 6, Figure 7, Figure 8, Figure 9, Figure 10, Figure 11 and Figure 12. The plots of regression compare AI-based outputs with numerical targets; the unity line (diagonal) stands for perfect convergence. This unity line is only a reference and shows where AI outputs would fall if they were an exact match to the targets.

### 4.2. Velocity Profiles $F^{\prime }\left(\eta \right)$ and $G^{\prime }\left(\eta \right)$ (Scenario S-1 to S-5)

Figure 6e and Figure 7e illustrate the variation of the velocity profiles $F^{\prime }\left(\eta \right)$ (horizontal velocity component) and $G^{\prime }\left(\eta \right)$ (vertical velocity component) with different values of the magnetic parameter *M*. Considered values are M=0.1,0,4,0.7,1.1. General observation is that enhancing *M* results in minoring the velocity profiles $F^{\prime }\left(\eta \right)$ and $G^{\prime }\left(\eta \right)$ or otherwise, the velocity profile increases as the magnetic parameter decreases. Qualitative, one observes an almost linear dependence on $G^{\prime }\left(\eta \right)$ (Figure 7e), while the effect on $F^{\prime }\left(\eta \right)$ (Figure 6e) is clearly non-linear. Here the separation between the different curves increases with augmentation in *M* before for larger values the curves harmonize/come together when η approaches to end of domain, to be stable. This widened velocity profile is caused by the strong surface tension gradient, thermal difference and interaction of nanoparticles within boundary layer. large parameter η diminishes the influence due to thermal diffusion, reducing forces and stabilizing particles interaction, leads to monotonous convergence. In addition values of $F^{\prime }\left(\eta \right)$ are about one order larger than $G^{\prime }\left(\eta \right)$. The origin of the decreasing behavior is the fact that a weaker magnetic field imposes less resistance to the fluid’s motion. The corresponding error plots (Figure 7f) for the differences between Python-generated and AI-generated outputs in the profiles differs from null values, which validates AI-results.

The influence of variation in Darcy numbers Da on the velocity profiles $F^{\prime }\left(\eta \right)$ (horizontal) and $G^{\prime }\left(\eta \right)$ (vertical) are presented in Figure 8 and Figure 9. Increasing Da causes the horizontal velocity profiles $F^{\prime }\left(\eta \right)$ to decrease (Figure 8e). With increasing η, one observes a general reducing velocity trend whereby the velocity gets closer to its minimum value of zero, which means that fluid slows down as it gets distant from the reference point. Also, the values of higher Da lead to the decreasing velocity of the fluid, proving absorbing hindrance in mobility of fluid. In contrast to this, the vertical velocity profiles $G^{\prime }\left(\eta \right)$ (Figure 9e) just behave in opposite manner. Here lower Darcy numbers Da diminish the vertical velocity profile $G^{\prime }\left(\eta \right)$ indicating that specific distribution of the velocity components suggests the preference to the vertical velocities under certain conditions. This increased value of the vertical velocity component for an increment of Da can be understood by the fact that higher Da is associated with lower flow resistance, hence, greater velocities, including in the vertical direction. For both velocity conponents $F^{\prime }\left(\eta \right)$ and $G^{\prime }\left(\eta \right)$, the relative value by which the curves are separated decreases and the degree of separation minimizes towards the end of the domain where the flow approaches a state of stasis or equilibrium.

Figure 10e illustrates (scenario S-5) how the vertical velocity profile $G^{\prime }\left(\eta \right)$ varies with η for different values of the velocity ratio parameter *S*. As *S* increases, the velocity difference between the free stream and the wall becomes more and more significant, leading to a steeper $G^{\prime }\left(\eta \right)$ gradient. This indicates stronger flow acceleration near the wall and increased shear effects within the boundary layer.

### 4.3. Temperature Profiles θ(η) (Scenario S-6 and S-7)

Increasing the thermal radiation coefficient (*R*) and the retardation parameter Rd has a similar effect on the temperature profile θ(η) as illustrated in Figure 11e and Figure 12e. The corresponding values of θ(η) become reduced.

Figure 11e illustrates that the decrease in the temperature profile θ(η) with variation in Rd is largest at 1.5≲η≲2. Larger values *R* cause a faster heat loss, leading to steeper temperature gradients, demonstrating that radiation significantly enhances cooling in the system.

The temperature profile θ(η) also become smaller with increasinng retardation parameter Rd as presented in Figure 12e. It holds a capacity to affect the heat retention of the system as it counteracts thermal decay. This means that when Rd is high heat dissipation is retained for a longer time inside the medium and near the subject surface and this results in high temperature gradient within the fluid. This is especially noticeable in the systems characterized with convective and radiative heat transfer modes.

## 5. Conclusions

This article centers on the study of THNF flow over a stretchable porous sheet, a phenomenon with significant applications in biomedical engineering and applied sciences. A Mathematica-based algorithm has been employed to produce a numerical dataset, complemented by AI-driven solution graphs analyzed through the Levenberg Marquardt Algorithm (LMFA) approach. Comprehensive comparison and training plots in various formats are presented, offering detailed insights into the behavior of THNF flow over stretchable skin.

Thus, as a general outcome of this work, it can be concluded that a Ternary-hybrid nanofluid can have useful applications in thermodynamic systems especially as it appears that the behavior of the manufactured fluid can be more effectively controlled. Therefore, the performance of Ternary-HNF is substantiated with great optimizing capability in heat and mass transfer, which has been revealed in this paper. Moreover, we have shown that considering machine learning (ML) techniques as novel approach with the use of LMFA to train neural network, produces cost and time efficient optimized results. This can be seen as a major step forward in academic advancement and practice with the simulations and predictions in intricate systems in general. We hope that our work will inspire other researchers, experimentalists, mathematics, and numerical simulations to work on the challenges of economics and stability, which can introduce extended opportunities for the line of product application.

## Figures and Tables

**Table 1:** Thermophysical properties of conventional liquid and nanoparticles.

Physical Properties	Blood	MWCNT	Gold	Silver
$c_{p}$ (J/kg K)	3617	796	129.1	235
ρ (kg/m^3^)	1050	1600	19,300	10,500
κ (W/mK)	0.52	3000	318	429
σ (S/m)	1090	105	4.52 × 10^7^	3.6 × 10^7^
φ	0.01	0.01	0.01	0.04

**Table 2:** Effective thermophysical parameters.

Parameter	Value
$\mu_{tnf}$	0.0045
$\rho_{tnf}$	1608.4272
$\sigma_{tnf}$	1247.1246
$c_{tnf}$	2305.4235
$k_{tnf}$	0.6206

**Table 3:** Numerical variations of parameters across various considered scenarios S-1 to S-7.

Scenarios	Cases	Parameters					
		M	Da	S	R	Pr	Rd
S-1	1	**0.1**	0.2	0.1	0.3	0.1	0.5
Variation	2	**0.4**	0.2	0.1	0.3	0.1	0.5
of *M* for	3	**0.7**	0.2	0.1	0.3	0.1	0.5
$F^{\prime }\left(\eta \right)$	4	**1.1**	0.2	0.1	0.3	0.1	0.5
S-2	1	**0.7**	0.2	0.1	0.3	0.1	0.5
Variation	2	**0.9**	0.2	0.1	0.3	0.1	0.5
of *M* for	3	**1.1**	0.2	0.1	0.3	0.1	0.5
$G^{\prime }\left(\eta \right)$	4	**1.3**	0.2	0.1	0.3	0.1	0.5
S-3	1	0.2	**0.3**	0.1	0.3	0.1	0.5
Variation	2	0.2	**0.6**	0.1	0.3	0.1	0.5
of Da for	3	0.2	**0.9**	0.1	0.3	0.1	0.5
$F^{\prime }\left(\eta \right)$	4	0.2	**1.2**	0.1	0.3	0.1	0.5
S-4	1	0.2	**0.3**	0.1	0.3	0.1	0.5
Variation	2	0.2	**0.6**	0.1	0.3	0.1	0.5
of Da for	3	0.2	**0.9**	0.1	0.3	0.1	0.5
$G^{\prime }\left(\eta \right)$	4	0.2	**1.2**	0.1	0.3	0.1	0.5
S-5	1	0.1	0.2	**0.8**	0.3	0.1	0.5
Variation	2	0.1	0.2	**1.0**	0.3	0.1	0.5
of *S* for	3	0.1	0.2	**1.2**	0.3	0.1	0.5
$G^{\prime }\left(\eta \right)$	4	0.1	0.2	**1.4**	0.3	0.1	0.5
S-6	1	0.1	0.5	0.8	**0.1**	2.5	0.9
Variation	2	0.1	0.5	0.8	**0.2**	2.5	0.9
of *R* for	3	0.1	0.5	0.8	**0.3**	2.5	0.9
θ(η)	4	0.1	0.5	0.8	**0.4**	2.5	0.9
S-7	1	0.1	0.5	0.8	0.2	1.0	**0.1**
Variation	2	0.1	0.5	0.8	0.2	1.0	**0.3**
of Rd for	3	0.1	0.5	0.8	0.2	1.0	**0.6**
θ(η)	4	0.1	0.5	0.8	0.2	1.0	**0.9**

**Table 4:** Mathematica Dataset Convergence Parameters.

Scenarios	M.S.E. Data			Grids	Gradient	Mu	Closing	T/s
	Trainung	Validation	Testing			Grids	Epoch	
	$10^{-10}$	$10^{-10}$	$10^{-10}$	$10^{-9}$	$10^{-8}$	$10^{-8}$		
S1	2.21	3.52	3.62	0.0002.21	9.97	1	432	0.1
S2	14.9	46.7	60.4	1.49	8.55	0.01	24	0.0
S3	2.09	3.51	3.43	2.09	9.95	1	411	0.1
S4	21.5	50.1	30.9	21.5	9.98	1	238	0.1
S5	1.49	14.1	2.14	1.49	9.95	1	208	0.1
S6	12.5	30.4	27.6	12.5	9.84	1	202	0.0
S7	1.07	1.55	2.01	1.07	10	0.1	620	0.1

## Data Availability

The raw data supporting the conclusions of this article will be made available by the authors on request.

## Abbreviations

The following abbreviations are used in this manuscript:

UCM	Upper-convected Maxwell
CNT	Carbon nanotubes
MHD	Magnetohydrodynamics
ML	Machine learning
ODE	Ordinary differential equation
PDE	Partial differential equation
LMFA	Levenberg-Marquardt Feedforward Algorithm
AI	Artificial intelligence

## Nomenclature

Parameter	Description	Parameter	Description
(*x*, *y*)	position coordinate	*Bi*	Biot value
*U_w_*	velocity along *x*-direction	*Pr*	Prandtl ratio
*V_w_*	velocity along *y*-direction	*S*	velocity ratio
*T_f_*	temperature of the fluid	*Da*	porosity characteristics
*B* _0_	Tesla value	*R*	radiation parameter
*K**	absorbing medium	*C_fx_*, *C_fx_*	local wall stresses
*Q* _0_	external heat	*Nu*	Nussel number
*q_r_*	radial flux	*Re*	Reynolds number
*T* _∞_	wall temperature	*Q*	heat source/skin characteristics
*h*	thermal exchange rate	*Rd*	Retardation factor
*μ*	dynamic viscosity	*nf*	nanofluid
*σ*	electrical conductivity	*hnf*	hybrid nanofluid
*ρc_p_*	heat capacity	*thnf*	Ternary hybrid nanofluid
*k*	heat conduction rate	*M*	magnetic parameter
